# Is serum matrix metalloproteinase 9 a useful biomarker in detection of colorectal cancer? Considering pre-analytical interference that may influence diagnostic accuracy

**DOI:** 10.1038/sj.bjc.6604491

**Published:** 2008-07-22

**Authors:** K Jung

**Affiliations:** 1 Department of Urology, University Hospital Charité and Berlin Institute for Urologic Research, Berlin, Germany

**Sir**,

I read with great interest the article by Hurst *et al* (2007), which was recently published in the *Br J Cancer*. The authors described elevated serum concentration of matrix metalloproteinase 9 (MMP-9) in patients with colorectal neoplasia compared with the MMP-9 concentration in symptomatic patients without non-neoplastic conditions. On the basis of this result of increased serum MMP-9 concentration the authors developed a logistic regression model including age, sex, smoking history, abdominal pain, and weight loss as additional factors for the prediction of malignant colorectal disease. The authors concluded that increased serum MMP-9 concentrations could be useful to stratify patients into those with low- and high-risk of malignancy to spare patients unnecessary colonoscopy. Some of the authors of this article already elaborated detailed study protocols including sample size calculation, potential selection bias, confounders, methods of analyses study protocols to evaluate the potential of serum MMP-9 as a screening test for colorectal cancer (Ryan *et al*, 2006; Wilson *et al*, 2006). Apparently based on the pilot data described in the mentioned report of Hurst *et al* (2007) and the prevalence data of colon cancer, it was calculated that 23 100 people from 29 practices have to be recruited to identify the 700 participants needed for this study (Wilson *et al*, 2006).

However, I have the impression that the authors overlooked the fundamental problem of pre-analytical conditions for accurate MMP-9 measurements. They did not pay attention to the potential pre-analytical pitfall of blood sampling to measure true concentrations of circulating MMP-9 as serum instead of plasma was used for MMP-9 measurements and also recommended for this ambitious study. The misuse of serum as sample for determining circulating MMP-9 in peripheral blood has been frequently criticised both in analytical and clinical journals (Jung *et al*, 1998; Makowski and Ramsby, 2003; Mannello *et al*, 2003, 2007; Meisser *et al*, 2005; Souza-Tarla *et al*, 2005; Zucker and Cao, 2005). In addition, technical details of serum sampling and handling procedure, for example the time between phlebotomy and centrifugation of blood samples, should be described because they are critical determinants that could affect the concentrations of MMP-9 (Jung *et al*, 2005; Meisser *et al*, 2005; Mannello and Tonti, 2007).

To illustrate that problem comparative MMP-9 measurements were performed in serum and plasma samples that were obtained under different collection conditions. For that purpose, blood samples from 10 healthy adults were simultaneously collected in differently prepared plastic tubes (Monovette Systems, Sarstedt AG, Nümbrecht, Germany) and centrifuged at 1600 **g** at 4°C for 15 min within 30 min after venepuncture. Tubes either with kaolin-coated granulate as clot activator or without additive were used to prepare serum after enhanced coagulation (serum^(+)^) and native serum (serum^(−)^), respectively whereas tubes coated with lithium heparin, sodium citrate or dipotassium EDTA were used to collect plasma samples. The supernatants were carefully removed and stored at −80°C until analysis. MMP-9 was measured in duplicates with the Fluorokine MultiAnalyte Profiling assay (R&D Systems, Minneapolis, MN, USA) detecting pro-, mature, and TIMP complexed MMP-9 according to the note of the producer. The coefficient of variation calculated from the duplicate values was 9.7%. The striking effect of sample collection on the MMP-9 concentrations is presented as percentage data related to values obtained in serum^(+)^ collected with clot activator (Figure 1). Higher MMP-9 concentrations were observed in serum than in plasma. In addition, the kind of serum sampling procedure with or without clot activator was important. The concentrations found in serum^(+)^ samples collected with clot activator were about 3–4 times higher compared with those in serum^(−)^ samples collected without clot activator. However, also the MMP-9 concentrations in serum^(−)^ samples collected without clot activator corresponding to serum collected in plain tubes using the Vacutainer system by Hurst *et al* (2007) were 3–5 times higher than in heparin or citrate plasma. All these data show that serum MMP-9 concentrations do not correspond to the real circulating concentrations of MMP-9 in blood because the anticoagulants do not affect the recovery of MMP-9 in samples (Jung *et al*, 1998). Platelets and leukocytes abundantly contain MMP-9 among other proteases (Murphy *et al*, 1977; Cooper *et al*, 1985; Opdenakker *et al*, 2001; Sheu *et al*, 2004; Santos-Martinez *et al*, 2008). The increased MMP-9 concentration observed in serum may be due both to release of MMP-9 during coagulation from the blood cells and its secretion that is induced by the clot activator itself (Mannello and Tonti, 2007; Mannello *et al*, 2007). Thus, it can be assumed that this high unspecific background concentration of MMP-9 in serum probably impairs the potential diagnostic/prognostic performance of this parameter. Recent studies confirmed that plasma MMP-9 had better diagnostic accuracy than serum MMP-9 (Jung *et al*, 2001; Wu *et al*, 2007). Also the consistent use of serum collected under identical conditions was obviously not suitable to circumvent that effect.

In conclusion, serum collected either with or without clot activator should not be considered as an appropriate sample for determining circulating MMP-9. Pre-analytical conditions of sample collection and handling have to be clarified before the diagnostic or prognostic performance of a corresponding marker should be explored in clinical trials (Mannello *et al*, 2005; Lomholt *et al*, 2007). It may be advisable to re-consider the sample collection procedure for the planned studies. Citrate plasma has been suggested to be the sample of choice for measuring circulating MMP-9 (Makowski and Ramsby, 2003; Mannello *et al*, 2003; Meisser *et al*, 2005; Gerlach *et al*, 2005).

## Figures and Tables

**Figure 1:**
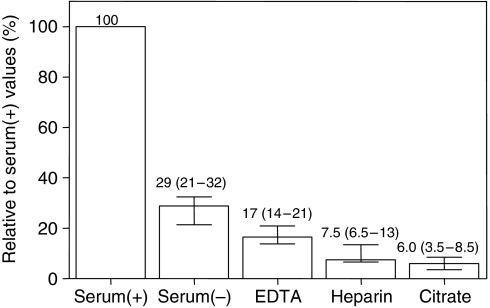
Relative concentrations of MMP-9 in serum and plasma samples. Percentage values (medians with interquartile ranges) of MMP-9 concentrations in serum and plasma samples simultaneously collected from 10 healthy adults were related to the values in serum^(+)^ samples (median: 536 *μ*g l^−1^; interquartile ranges: 384–692 *μ*g l^−1^) that were taken as 100%. Further details see text. Serum^(+)^ and serum^(−)^: serum prepared with and without clot activator (kaolin-coated granulate).

## References

[bib1] Cooper TW, Eisen AZ, Stricklin GP, Welgus HG (1985) Platelet-derived collagenase inhibitor: characterization and subcellular localization. Proc Nat Acad Sci USA 82: 2779–2783 2986137 10.1073/pnas.82.9.2779 PMC397649

[bib2] Gerlach RF, Uzuelli JA, Souza-Tarla CD, Tanus-Santos JE (2005) Effect of anticoagulants on the determination of plasma matrix metalloproteinase (MMP)-2 and MMP-9 activities. Anal Biochem 344: 147–149 15950912 10.1016/j.ab.2005.04.038

[bib3] Hurst NG, Stocken DD, Wilson S, Keh C, Wakelam MJ, Ismail T (2007) Elevated serum matrix metalloproteinase 9 (MMP-9) concentration predicts the presence of colorectal neoplasia in symptomatic patients. Br J Cancer 97: 971–977 17912241 10.1038/sj.bjc.6603958 PMC2360395

[bib4] Jung K, Laube C, Lein M, Lichtinghagen R, Tschesche H, Schnorr D, Loening S (1998) Kind of sample as preanalytical determinant of matrix metalloproteinases 2 and 9 (MMP2; MMP9) and tissue inhibitor of metalloproteinase 2 (TIMP2) in blood. Clin Chem 44: 1060–1062 9590387

[bib5] Jung K, Lein M, Laube C, Lichtinghagen R (2001) Blood specimen collection methods influence the concentration and the diagnostic validity of matrix metalloproteinase 9 in blood. Clin Chim Acta 314: 241–244 11718702 10.1016/s0009-8981(01)00679-9

[bib6] Jung K, Meisser A, Bischof P (2005) Blood sampling as critical preanalytical determinant to use circulating MMP and TIMP as surrogate markers for pathological processes. Int J Cancer 116: 1000–1001 15856460 10.1002/ijc.21129

[bib7] Lomholt AF, Frederiksen CB, Christensen IJ, Brunner N, Nielsen HJ (2007) Plasma tissue inhibitor of metalloproteinases-1 as a biological marker? Pre-analytical considerations. Clin Chim Acta 380: 128–132 17328880 10.1016/j.cca.2007.01.022

[bib8] Makowski GS, Ramsby ML (2003) Use of citrate to minimize neutrophil matrix metalloproteinase-9 in human plasma. Anal Biochem 322: 283–286 14596841 10.1016/j.ab.2003.07.030

[bib9] Mannello F, Luchetti F, Canonico B, Papa S (2003) Effect of anticoagulants and cell separation media as preanalytical determinants on zymographic analysis of plasma matrix metalloproteinases. Clin Chem 49: 1956–1957 14578336 10.1373/clinchem.2003.022145

[bib10] Mannello F, Tonti G, Papa S (2005) Matrix metalloproteinase inhibitors as anticancer therapeutics. Curr Cancer Drug Targets 5: 285–298 15975049 10.2174/1568009054064615

[bib11] Mannello F, Tonti GA (2007) Gelatinase concentrations and zymographic profiles in human breast cancer: matrix metalloproteinases circulating in plasma are better markers for the subclassification and early prediction of cancer: the coagulation/fibrinolysis pathways alter the release, activation and recovery of different gelatinases in serum. Int J Cancer 121: 216–218 17315186 10.1002/ijc.22652

[bib12] Mannello F, Tonti GA, Tanus-Santos JE, Gerlach RF (2007) Silicate increases the release of MMP-9 forms in peripheral blood: why gelatin zymography differs significantly in citrate plasma and serum obtained with or without clot activators. Clin Chem 53: 1981–1982 17954502 10.1373/clinchem.2007.090548

[bib13] Meisser A, Cohen M, Bischof P (2005) Concentrations of circulating gelatinases (matrix metalloproteinase-2 and -9) are dependent on the conditions of blood collection. Clin Chem 51: 274–276 15613733 10.1373/clinchem.2004.041707

[bib14] Murphy G, Reynolds JJ, Bretz U, Baggiolini M (1977) Collagenase is a component of the specific granules of human neutrophil leucocytes. Biochem J 162: 195–197 192209 10.1042/bj1620195 PMC1164583

[bib15] Opdenakker G, Van den Steen PE, Dubois B, Nelissen I, Van CE, Masure S, Proost P, Van DJ (2001) Gelatinase B functions as regulator and effector in leukocyte biology. J Leukoc Biol 69: 851–859 11404367

[bib16] Ryan AV, Wilson S, Wakelam MJ, Warmington SA, Dunn JA, Hobbs RF, Martin A, Ismail T (2006) A prospective study to assess the value of MMP-9 in improving the appropriateness of urgent referrals for colorectal cancer. BMC Cancer 6: 251 17059590 10.1186/1471-2407-6-251 PMC1635060

[bib17] Santos-Martinez MJ, Medina C, Jurasz P, Radomski MW (2008) Role of metalloproteinases in platelet function. Thromb Res 121: 535–542 17681591 10.1016/j.thromres.2007.06.002

[bib18] Sheu JR, Fong TH, Liu CM, Shen MY, Chen TL, Chang Y, Lu MS, Hsiao G (2004) Expression of matrix metalloproteinase-9 in human platelets: regulation of platelet activation in *in vitro* and *in vivo* studies. Br J Pharmacol 143: 193–201 15289295 10.1038/sj.bjp.0705917 PMC1575278

[bib19] Souza-Tarla CD, Uzuelli JA, Machado AA, Gerlach RF, Tanus-Santos JE (2005) Methodological issues affecting the determination of plasma matrix metalloproteinase (MMP)-2 and MMP-9 activities. Clin Biochem 38: 410–414 15820769 10.1016/j.clinbiochem.2005.02.010

[bib20] Wilson S, Wakelam MJ, Hobbs RF, Ryan AV, Dunn JA, Redman VD, Patrick F, Colbourne L, Martin A, Ismail T (2006) Evaluation of the accuracy of serum MMP-9 as a test for colorectal cancer in a primary care population. BMC Cancer 6: 258 17076885 10.1186/1471-2407-6-258 PMC1654179

[bib21] Wu CY, Wu MS, Chiang EP, Chen YJ, Chen CJ, Chi NH, Shih YT, Chen GH, Lin JT (2007) Plasma matrix metalloproteinase-9 level is better than serum matrix metalloproteinase-9 level to predict gastric cancer evolution. Clin Cancer Res 13: 2054–2060 17404086 10.1158/1078-0432.CCR-06-2299

[bib22] Zucker S, Cao J (2005) Measurement of matrix metalloproteinases in serum of patients with melanoma: snarled in technical pitfalls. Clin Cancer Res 11: 5069–5070 16033818 10.1158/1078-0432.CCR-05-0774

